# Molecular mechanism of Danshen injection in treating endometrial fibrosis induced by intrauterine adhesions via the LAMC2-CD44-TGF-β1-SMAD2/3 signaling pathway

**DOI:** 10.3389/fphys.2026.1794215

**Published:** 2026-04-10

**Authors:** Sisi Tang, Renzhi Hu, Shuangyan Weng, Mei Luo

**Affiliations:** Gynecological Reproductive Center, Chongqing Traditional Chinese Medicine Hospital, Chongqing, China

**Keywords:** Danshen injection, fibrosis, intrauterine adhesion, molecular mechanism, TGF-β1- SMAD2/3 signaling pathway

## Abstract

**Introduction:**

Danshen injection, derived from the traditional Chinese medicine *Salvia miltiorrhiza* Bunge, has been clinically validated for its safety and efficacy in treating various gynecological diseases, including endometrial fibrosis caused by Intrauterine Adhesion (IUA). However, its molecular mechanism in regulating the Laminin Subunit Gamma 2 (LAMC2)-CD44-TGF-β1-SMAD2/3 signaling pathway remains unclear.

**Methods:**

An IUA rat model was established and treated with low-, medium-, or high-dose Danshen injection via tail vein injection. Endometrial injury and fibrosis were assessed by H&E and Masson’s staining, and epithelial/mesenchymal markers were evaluated by immunohistochemistry and Western blot. Single-cell RNA sequencing (scRNA-seq) was performed to characterize cellular composition, pathway enrichment, and predicted intercellular communication. *In vitro*, Lipopolysaccharide (LPS)-stimulated uterine fibroblasts were used to examine LAMC2 expression, Epithelial-Mesenchymal Transition (EMT)-related marker changes, and TGF-β1/Smad signaling, with pathway interrogation using a CD44 blocker. Simultaneously, corresponding verification was conducted *in vivo*.

**Results:**

Danshen injection improved uterine histopathology, increased endometrial gland number, and reduced collagen deposition in IUA rats. CK-19 and Vimentin showed changes consistent with epithelial–mesenchymal marker alterations, which were partially reversed after Danshen treatment. scRNA-seq revealed an increased fibroblast proportion in the Model group that decreased after Danshen intervention, with differentially expressed genes enriched in fibrosis-related pathways. Cell–cell communication analysis suggested reduced repair-associated signaling and increased LAMC2-associated interactions in the Model group. *In vitro*, LPS induced LAMC2 upregulation and activation of TGF-β1/SMAD2/3 signaling, which was attenuated by CD44 blockade; similarly, Danshen injection and CD44 inhibition reduced LAMC2 and TGF-β1/SMAD2/3 activation *in vivo*.

**Conclusion:**

Danshen injection may mitigate endometrial fibrosis in IUA, potentially by modulating fibroblast abundance, reducing EMT-related changes, and dampening LAMC2-CD44-associated TGF-β1/SMAD2/3 signaling.

## 1 Introduction

Intrauterine Adhesion (IUA), a prevalent gynecological condition, is defined by the partial or total adhesion within the uterine cavity (Munro et al., 2025). This pathological process arises from impaired healing of endometrial basal layer injuries, which may result from factors such as intrauterine interventions, infections, or inflammatory responses (Lee et al., 2021). Clinically, patients frequently exhibit symptoms including hypomenorrhea or amenorrhea, dysmenorrhea, infertility, recurrent pregnancy loss, and severe placental complications, thereby positioning it as a critical uterine etiology of infertility among reproductive-aged women (Deans and Abbott, 2010). In recent decades, the incidence of IUA has exhibited a significant increase, primarily attributed to the escalating adoption of hysteroscopic techniques and various uterine surgical procedures. Epidemiological investigations have revealed that the prevalence of IUA linked to repeated induced abortions or uterine curettage exceeds 20% (Deans and Abbott, 2010). Beyond surgical trauma, a subset of cases has been associated with endometrial tuberculosis, schistosomiasis, or congenital Müllerian duct malformations (Humphries and Isaacson, 2025).

Endometrial fibrosis serves as the core pathological mechanism underlying the onset and progression of IUA, which is essentially characterized by the abnormal activation of endometrial stromal fibroblasts, excessive deposition of extracellular matrix (ECM), and an imbalanced inflammatory microenvironment (Xu C. et al., 2022). LAMC2, a pivotal component of the basement membrane, is involved in cell-matrix adhesion and signal transduction. CD44, a transmembrane glycoprotein, not only mediates cell migration and proliferation but also regulates the fibrotic process by activating downstream pathways such as Janus kinase 2/Signal transducer and activator of transcription 3 (JAK2/STAT3), phosphatidylinositol 3-kinase (PI3K)/Akt, and mitogen-activated protein kinase (MAPK) (Guo et al., 2024; Yang Y. et al., 2025). Researchers have discovered through database analysis that the expression of LAMC2 in breast cancer is positively correlated with the tumor stem cell markers CD44 and CD133 (Zhao et al., 2023). However, the specific mechanisms of action of these two molecules in endometrial fibrosis associated with IUA remain to be elucidated.

Currently, the primary clinical treatment for IUA is transcervical resection of adhesions (TCRA); however, postoperative re-adhesion remains common, especially in moderate-to-severe disease (AAGL Elevating Gynecologic Surgery, 2017). Hormonal therapy (e.g., estrogen), intrauterine devices, or biomaterials are often used as adjuncts to prevent recurrence, but the effectiveness of these strategies remains limited and may be associated with side effects (Vitale et al., 2022). Exploring effective and safe anti-adhesion drugs has become an important research direction. Therefore, it is imperative to identify new therapeutic strategies, and understanding the pathogenesis of IUA is crucial for developing innovative treatment approaches.

In this context, traditional Chinese medicine (TCM) has demonstrated promising potential. Danshen injection, a widely utilized TCM injection employed for invigorating blood circulation and resolving blood stasis, contains bioactive components such as Tanshinone IIA and demonstrates antifibrotic and antiadhesive properties (Xu et al., 2016, Tanshinone IIA). Its mechanisms may involve improving microcirculation, activating the fibrinolytic system, promoting fibrinolysis, inhibiting excessive fibroblast proliferation and collagen synthesis, and potentially regulating signaling pathways such as TGF-β1/Smad, platelet-derived growth factor (PDGF), and tissue inhibitor of metalloproteinase-1 (TIMP-1) (Ding et al., 2012; Xu WJ. et al., 2022). However, whether Danshen injection exerts inhibitory effects on endometrial fibrosis via modulation of the specific LAMC2-CD44-TGF-β1-SMAD2/3 signaling axis has not been thoroughly investigated, and the precise molecular underpinnings remain to be clarified. Therefore, the present study is designed to explore the molecular mechanisms underlying the therapeutic effect of Danshen injection on endometrial fibrosis in IUA, with a special emphasis on corroborating the pivotal role of the LAMC2-CD44-TGF-β1-SMAD2/3 signaling cascade. The findings will provide a theoretical basis for the clinical application of Danshen injection and offer new molecular targets and therapeutic strategies for the prevention and treatment of IUA.

## 2 Materials and methods

### 2.1 Experimental animals

98 female Sprague-Dawley (SD) rats, Specific Pathogen-Free (SPF)grade, aged 8–10 weeks, were supplied by Chengdu Dashuo Experimental Animal Co., Ltd. All rats were raised under the same conditions, including temperature maintained at 23 ± 2 °C, humidity maintained at 35-60%, and the animal room following a 12 h diurnal/nocturnal cycle.

### 2.2 IUA model establishment and experimental animal grouping

The steps for constructing the rat intrauterine adhesion model were presented as follows: Firstly, harnessing the vaginal smear method, we identified rats in estrus for model construction. Prior to surgery, water intake was restricted for 8–12 h. Anesthesia was induced via chloral hydrate, and the rats were placed in a supine position on the surgical table for animals. After skin preparation and alcohol disinfection of the lower abdomen, a vertical incision was made 2 finger-widths above the pubic symphysis. Subsequently, the uterus was exposed layer by layer, and a 0.5 cm vertical incision was made in the lower 1/3 of the uterus. A 25G sterile injection needle was selected, and its tip was flattened and blunted using a sterile grinding tool to prevent sharp ends from penetrating the uterine wall. The needle tip was gently bent into a small arc-shaped blunt tip (arc angle ≈ 15°) to match the uterine cavity morphology of rats, facilitating gentle endometrial curettage. During the experiment, intrauterine curettage was performed solely on the right uterine horn to induce IUA lesions, while the left uterine horn remained intact without any damage or manipulation. A lipopolysaccharide (L861706–1 mg, Macklin, China) cotton thread was placed in the injured segment of the uterus, with about 3 cm left and the excess thread cut off, followed by layer-by-layer suturing. After rinsing the abdominal cavity, the surgery was concluded. Throughout the entire surgical process, strict aseptic principles were adhered to. Two days later, the cotton thread inside the uterine cavity was pulled out (Xu C. et al., 2022).

50 animals were used for efficacy validation, and 48 animals were used for mechanism validation. The specific grouping information is shown in **Tables 1**, **2**.

**Table 1:** Animal grouping for efficacy verification.

Group	n	Administration dosage	Main readouts
Sham	10	——	H&E/Masson/IHC, WB, Single cell sequencing
Model	10	——	H&E/Masson/IHC, WB, Single cell sequencing
Model+L-Danshen injection	10	0.6 mL/kg i.v.	H&E/Masson/IHC, WB,
Model+M-Danshen injection	10	1.2 mL/kg i.v.	H&E/Masson/IHC, WB
Model+H-Danshen injection	10	2.4 mL/kg i.v.	H&E/Masson/IHC, WB, Single cell sequencing

**Table 2:** Animal grouping for mechanism verification.

Group	n	Administration dosage	Main readouts
Sham	12	——	H&E/Masson, WB, IF
Model	12	——	H&E/Masson, WB, IF
Model+ Danshen injection	12	1.2 mL/kg i.v.	H&E/Masson, WB, IF
Model+ CD44 inhibitor	12	5 mL/kg i.v.	H&E/Masson, WB, IF

‘,’ indicates the use of tissue samples alone (n=3); ‘/’ represents shared organizational samples (n=3); The remaining organizational samples will be kept for experimental emergency use.

Drug administration: Danshen injection or saline was administered via tail vein injection on postoperative days 7, 10, and 13. Danshen injection doses were 0.6, 1.2, and 2.4 mL/kg (low/medium/high), with saline volume matched to sham and model group (2.4 mL/kg). The CD44 inhibitor doses were 5 mL/kg (GTX76383, GeneTex, USA).

### 2.3 Primary cell isolation, culture, and treatment preparation

Primary rat endometrial fibroblasts were isolated from the healthy, unmanipulated uterine horns of 8-week-old female Sprague-Dawley rats to ensure physiological relevance. Briefly, the excised uterine tissues were thoroughly washed in ice-cold PBS, longitudinally opened, and minced into fine fragments of approximately 1-mm³. The tissue fragments were then subjected to enzymatic dissociation utilizing a highly optimized solution of 0.1% Type I collagenase (Gibco, USA) dissolved in HBSS, incubated at 37 °C for 60 minutes with gentle mechanical agitation.

Following digestion, the resulting heterogeneous cell suspension was filtered through a 70-μm sterile nylon mesh strainer to eliminate large, undigested tissue debris and glandular epithelial clusters. To achieve a highly purified population of fibroblasts, we exploited the differential adhesion time method. The filtered cell suspension was seeded into 25-cm² culture flasks containing Complete Medium (DMEM/F12 supplemented with 10% Fetal Bovine Serum and 1% penicillin-streptomycin) and incubated at 37 °C in a humidified 5% CO_2_ atmosphere. Given that stromal fibroblasts attach to plastic surfaces significantly faster than epithelial cells, the culture medium containing unattached epithelial cells and debris was aspirated and discarded after precisely 2 h. Fresh medium was added to the adherent fibroblasts. The medium was subsequently replaced every 48 h. To guarantee phenotypic stability and prevent replicative senescence, only primary fibroblasts from passages 2 through 4 were utilized for all subsequent *in vitro* pharmacological interventions. Endometrial epithelial cells purchased from Prenosat (CP-R049).

For the *in vitro* stimulation assays, 10 mg of LPS (HY-D1056, MCE, USA) was added to 10 mL of sterile water and completely dissolved to prepare a 1 mg/mL LPS solution. Subsequently, the solution was aliquoted and stored at -20 °C in a refrigerator for subsequent use. Next, 20 μL of the 1 mg/mL LPS solution was precisely pipetted and diluted to a final volume of 10 mL in complete medium alone. Following thorough mixing, complete medium with a final LPS concentration of 2 μg/mL was prepared and reserved for later use (Guo et al., 2019). In a similar manner, 80 μL of a 500 μg/mL CD44 solution was accurately pipetted and diluted to a final volume of 4 mL in complete medium alone. After adequate mixing, complete medium with a final CD44 concentration of 10 μg/mL was prepared and kept aside for subsequent use (Wu et al., 2019). The experimental procedures were conducted as follows: Cells were seeded into corresponding culture plates at an appropriate density and incubated at 37 °C in a humidified atmosphere with 5% CO_2_. Typically 2–4 days after seeding, cells reached 80-85% confluence, and subsequent treatment involved assigning cells to different intervention groups. The groups were configured as follows: the normal control group, where cells were maintained in complete medium with rat endometrial epithelial cells; the LPS-stimulated group, where cells were exposed to complete medium containing 2 μg/mL LPS for 24 h; and the LPS + CD44 inhibitor group, where cells were simultaneously treated with complete medium containing 2 μg/mL LPS and 30 μg/mL CD44 inhibitor for 24 h. After treatment completion, the cells were harvested for subsequent assays.

### 2.4 Parameter determination of endometrium in rats

Rats were euthanized on day 16 after model induction. Body weight was recorded at days 3, 6, 9, 12, and 15. Uterine length (from the cervix to the distal end of the horn) was measured using a digital caliper, and uterine wet weight was measured after removal of excess connective tissue.

Adhesion grading: The injured uterine horn was opened longitudinally and adhesions were graded macroscopically by two blinded observers using a four-level scale adapted from commonly used hysteroscopic severity criteria, based on the extent of cavity occlusion: Grade 1 (<25%, filmy/focal adhesions), Grade 2 (25–50%), Grade 3 (50–75%), and Grade 4 (>75% or near-complete obliteration) (Mazuji et al., 1964).

Histology: Uterine tissues were fixed in 4% paraformaldehyde for 24 h, embedded in paraffin, sectioned at 4 µm, and stained with H&E and Masson’s trichrome. Three visual fields per animal were randomly selected under 20× and 40× magnification, and the number of endometrial glands and collagen volume fraction were statistically analyzed by ImageJ.

### 2.5 Immunohistochemistry (CK-19 and vimentin)

Tissue sections were dewaxed and subjected to antigen retrieval using antigen extraction solution (G1201, Servicebio, China). After blocking with 5% goat serum (C0265, Beyotime, China) for 0.5 h at room temperature, sections were incubated with CK-19 (1:400; YM8269, Immunoway, USA) and vimentin (1:1000; YM8324, Immunoway, USA) diluted in 5% goat serum overnight at 4°C. Following washing, sections were incubated with a rabbit anti-IgG secondary antibody (1:500; GB23303, Servicebio, China) for 1.5 h at room temperature. 3,3’-Diaminobenzidine (DAB) (ZLI-9019, ZSBIO, China) staining was performed on sections in the dark for 1 min, followed by a 5-min washing with tap water. Sections were then counterstained with hematoxylin (G1004, Servicebio, China) for 1 min, differentiated using 1% HCl in ethanol for 2 s, followed by decolorization with tap water for a duration of 10 min. Following dehydration in 95% ethanol twice for 10 min each time, sections were cleared in xylene twice for 5 min each time and mounted with neutral resin (10004160, SINOPHARM, China). Images were acquired using an Mshot MF53 inverted microscope.

Immunohistochemical optical density quantification in this study utilized three parameters: positive staining area (AREA), integrated optical density (IOD), and average optical density (AOD). The computational procedure involved: digital scanning of immunostained sections via ImageJ software, selection of three random fields for measurement of AREA and cumulative IOD, followed by calculation of unit area protein expression intensity using the formula AOD = IOD/AREA. Bar graphs were constructed using mean AOD values per experimental group, with error bars representing Standard Error of the Mean (SEM).

### 2.6 Immunofluorescence staining (COL1A1, COL3A1, and α-SMA)

After deparaffinization and antigen retrieval, sections were permeabilized with 0.1% Triton X-100 (10 min) and blocked with 5% goat serum (30 min). Sections were incubated overnight at 4 °C with primary antibodies against COL1A1 (1:200; A27820, ABclonal, China), COL3A1 (1:100; A3795, ABclonal, China), and α-SMA (1:100; A7248, ABclonal, China), followed by fluorophore-conjugated secondary antibodies (1 h, room temperature, in the dark, 1:200; GB21303, Servicebio, China). Nuclei were counterstained with 4’,6-Diamidino-2-Phenylindole (DAPI), and sections were mounted with antifade medium. Fluorescence images were acquired using the same exposure settings within each experiment. Mean fluorescence intensity was quantified in ImageJ from matched regions of interest, with background subtraction.

### 2.7 ELISA detection of LAMC2 expression in endometrial epithelial cells

This assay employs a double-antibody sandwich ELISA to quantify LAMC2 (RA22147, Wuhan Beinerlai Biotechnology, China) levels in endometrial epithelial cells. Initially, culture supernatants were collected, centrifuged (3,000×g, 20 min, 4 °C) to remove debris, and stored as single-use aliquots at −80 °C. For cellular expression, endometrial epithelial cells were washed with ice-cold PBS and lysed in RIPA buffer (50 mM Tris-HCl, pH 7.4; 150 mM NaCl; 1% NP-40; 0.5% sodium deoxycholate; 0.1% SDS) containing a protease inhibitor cocktail. Lysates were clarified (3,000×g, 20 min, 4 °C), and total protein was quantified by Bicinchoninic Acid (BCA) assay; samples were normalized with kit diluent to 1 mg/mL and run in duplicate. Standards were prepared by serial two-fold dilution (0–800 pg/mL). In pre-coated 96-well plates, 40 µL of sample was added plus 10 µL biotinylated anti-LAMC2 antibody, followed by the addition of 50 µL HRP conjugate (blanks received diluent only). Plates were incubated at 37 °C for 30 min, washed five times, developed with substrates A/B (50 µL each) for 10 min in the dark, stopped (50 µL), and read at 450 nm within 15 min; concentrations were interpolated from the standard curve (10–800 pg/mL).

### 2.8 Western blot

Uterine tissues or cultured cells were lysed in RIPA buffer containing protease and phosphatase inhibitors. Protein concentrations were measured by BCA assay. After the total protein was quantified by BCA to 500 μg, it was mixed with 5× SDS loading buffer at a volume ratio of 1:4, supplemented with ddH_2_O to a final volume of 40 μL to form a 1× working solution. After heating at 95 °C for 10 min to achieve complete denaturation, 4.8 μL (containing 60 μg protein, calculated as 500 μg/40 μL × 4.8 μL) was taken for SDS-PAGE electrophoresis, and then transferred to PVDF membrane for immunoassay. Membranes were blocked in 5% Bovine Serum Albumin (BSA) for 1 h at room temperature and incubated with primary antibodies overnight at 4 °C, followed by HRP-conjugated secondary antibodies (1:2000, 1 h, room temperature). Bands were visualized using ECL and quantified in ImageJ. Total protein signals were normalized to GAPDH, and phosphorylation was expressed as the ratio of phospho/total SMAD2 or SMAD3. Densitometric analysis of Western Blot (WB) bands via ImageJ software to quantify the grayscale values of the target protein and the reference protein (GAPDH). Relative expression= target protein grayscale value/GAPDH grayscale value, yielding the expression intensity per unit of reference protein. Bar graphs were generated using the mean relative expression values across experimental groups, with error bars representing the standard deviation (SD).

Primary antibodies: CK-19 (1:1000; YM8269, Immunoway, USA), Vimentin (1:1000; YM8324, Immunoway, USA), GAPDH (1:1000; A19056, ABclonal, China), LAMC2 (1:1000; A1869, ABclonal, China), E-cad (1:1000; A20798, ABclonal, China), N-cad (1:500; A0433, ABclonal, China), α-SMA (1:1000; A2319, ABclonal, China), TGF-β1 (1:2000; A15103, ABclonal, China), SMAD2 (1:5000; bsm-52223R, Bioss, China), SMAD3 (1:1000; A16913, ABclonal, China), p- SMAD2 (1:5000; bs-3419R, Bioss, China), p- SMAD3 (1:2000; Ap0727, ABclonal, China), and a secondary antibody (1:2000; AS014, ABclonal, China).

### 2.9 Single cell sequencing analysis

Single-cell RNA sequencing was performed on uterine tissue from three groups: sham-operated controls, saline-injected model controls, and Danshen high-dose (2.4 mL/kg) treated rats. Tissue processing involved ice-cold Dulbecco’s Phosphate-Buffered Saline (DPBS) washing, mechanical dissociation, enzymatic digestion (37 °C, 20 min), and filtration through a 40 µm cell strainer. After erythrocyte lysis and viability staining (>80% by Trypan Blue), cells were processed via 10X Genomics.

Data quality control was implemented using Seurat (v4.3.0). Cells were filtered based on gene count (200–7500), mitochondrial gene content (<20%), and hemoglobin gene proportion (<5%). Batch effects were corrected using Harmony (v1.0). Dimensionality reduction and clustering were performed via Uniform Manifold Approximation and Projection (UMAP), with clusters visualized using ggplot2. Cell types were annotated using SingleR (v1.12.0) with reference to the ImmGen dataset. Differential gene expression analysis utilized the MAST algorithm, with results visualized through volcano plot (ggplot2) and heatmaps (pheatmap). Functional enrichment analysis employed ClusterProfiler (v4.4.4) for Gene Ontology (GO) terms and KEGG pathway analysis, with significant terms (p.adj < 0.05) displayed via bar charts and bubble plots.

To decipher the complex intercellular signaling networks within the endometrial microenvironment, cell-cell communication analysis was systematically executed using the CellChat R package (v1.6.1). By integrating the comprehensive CellChatDB database, which encompasses validated ligand-receptor multisubunit complexes, we inferred cell-state-specific communication networks. The signaling communication probabilities were quantitatively modeled utilizing a mass-action-based framework applied to the normalized scRNA-seq gene expression matrices. To identify statistically significant ligand-receptor interactions across the specific interacting cell groups, rigorous permutation tests were conducted with a significance threshold set at *P* < 0.05. Global communication patterns were evaluated to detect overarching topological shifts in signaling information flow. Crucially, to dissect the pharmacological mechanisms of Danshen injection, differential communication network analyses were meticulously performed across three defined pairwise comparisons: Model vs. Sham (to identify pathogenic signaling), Treat vs. Model (to evaluate treatment-induced suppression of pathogenic signaling).

### 2.10 Statistical analysis

For macro-morphological assessments (including uterine weight/length and adhesion grading), which are operationally convenient yet exhibit relatively large coefficients of variation, the right uterine horns from 6 independent animals per group were allocated for testing. In contrast, for high-throughput or high-cost molecular histological assays (such as Western blot, immunofluorescence, H&E/Masson staining, and scRNA-seq), the right uterine horns were randomly selected from 3 animals per group for analysis.

The experimental datasets were generated using GraphPad Prism 8 and expressed as mean ± standard deviation (***X¯* ± *s***). One-way analysis of variance (ANOVA) followed by *post-hoc* Tukey’s test was employed for multi-group comparisons. Differences were considered statistically significant when *P* < 0.05, as determined by the ANOVA results.

## 3 Results

### 3.1 Danshen injection ameliorates endometrial fibrosis in rats with IUA

**Figure 1:**
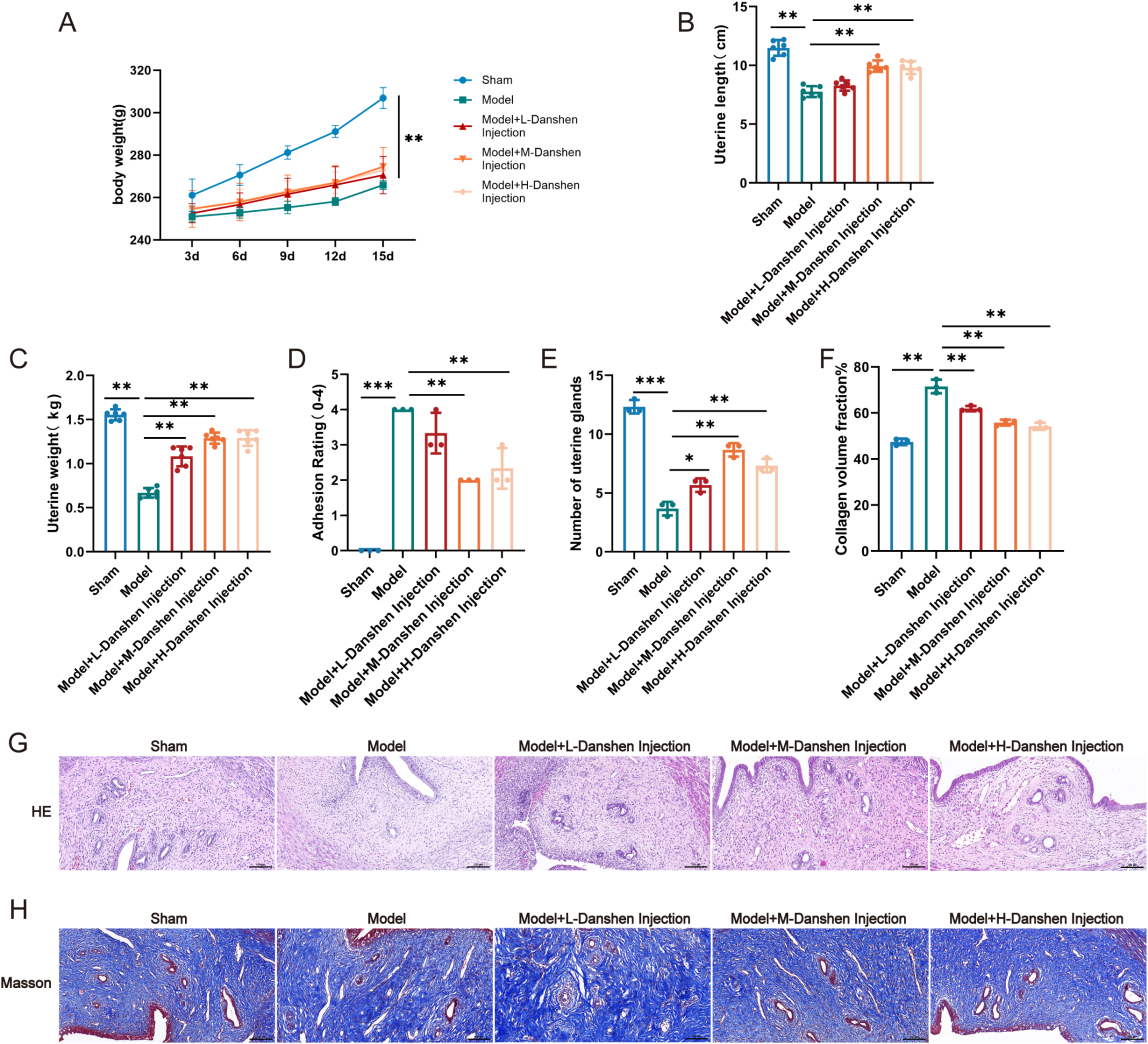
Danshen injection improves intrauterine adhesions in IUA rats. **(A)** Changes in body weight in each group, **(B)** Uterine Length, **(C)** Uterine Weight, **(D)** Grading of intrauterine adhesions in each group, **(E)** Number of uterine glands, **(F)** Collagen volume fraction, **(G)** H&E Staining, **(H)** MASSON Staining. Compared to model, *P < 0.05, **P < 0.01, ***P < 0.01. For panels A-C, n = 6 independent animals per group; for panels D-H, n=3 independent animals per group.

To evaluate the therapeutic effect of Danshen injection on endometrial fibrosis in IUA rats, we established an IUA rat model and administered low-, medium-, and high-dose Danshen injections via tail vein injection for treatment. The treatment protocol is illustrated in **Figure 1A**. Compared with the Sham group, Model rats exhibited significantly lower body weight, uterine length, and uterine weight, accompanied by pronounced uterine cavity narrowing and aggravated adhesion severity. Following Danshen injection intervention, these indicators showed significant reversal, particularly in the medium- and high-dose groups, where uterine adhesion grading decreased markedly, suggesting a restorative effect of Danshen injection on uterine structural damage induced by IUA (**Figures 1B–E**).

Further histological staining was performed to assess endometrial structure and the degree of fibrosis. H&E staining revealed that the Sham group had an intact endometrial structure with abundant and regularly arranged glands; in contrast, the Model group showed a significant reduction in gland number. After Danshen injection treatment, the gland count increased (**Figures 1F, H**). Masson staining results indicated extensive dense collagen fiber deposition in the endometrium of the Model group, with a fibrosis area proportion as high as 70%. In contrast, Danshen injection intervention significantly reduced collagen deposition and markedly decreased the fibrosis area (**Figures 1G, I**), suggesting its inhibitory effect on excessive collagen accumulation.

**Figure 2:**
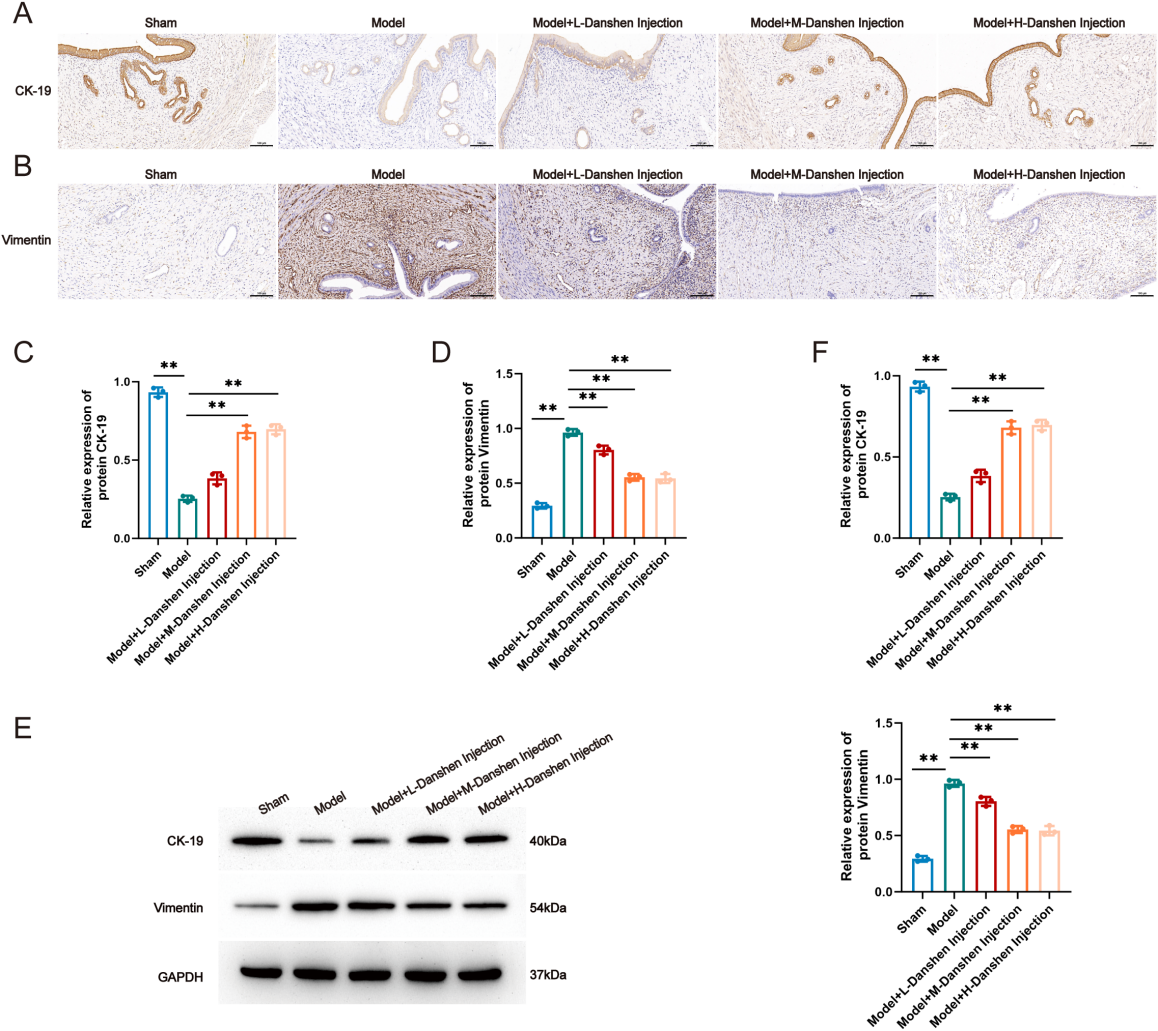
Danshen injection Improves Endometrial Fibrosis in IUA Rats. **(A)** Immunohistochemistry detection of CK-19 expression levels, **(B)** Immunohistochemistry detection of Vimentin expression levels, **(C)** Average optical density CK19, **(D)** Average optical density Vimentin, **(E)** WB detection of CK-19 and vimentin expression levels, **(F)** Relative expression levels of the CK-19 and vimentin proteins. Compared to model, *P < 0.05, **P < 0.01. For panels A-F, n=3 independent animals per group.

Additionally, we examined the expression of the epithelial marker CK-19 and the mesenchymal marker Vimentin in the endometrium using immunohistochemistry and WB assays. CK-19 expression was significantly reduced, whereas Vimentin expression was increased in the Model group. Danshen injection treatment, particularly in the medium- and high-dose groups, partially restored CK-19 expression and reduced Vimentin upregulation (**Figures 2A–F**), suggesting a potential role in preserving epithelial characteristics and limiting abnormal mesenchymal cells activation. A dose-dependent trend was observed between the low- and high-dose groups, while no significant difference was detected between the medium- and high-dose groups, suggesting that the medium dose may achieve a near-maximal effect under the current experimental conditions.

In summary, Danshen injection improved endometrial tissue structure and reduced collagen deposition in IUA rats. These findings suggest that it may alleviate endometrial fibrosis by helping preserve epithelial features and attenuating mesenchymal cells activation.

### 3.2 Single-cell sequencing reveals the role of fibroblasts in intrauterine adhesion in rats

**Figure 3:**
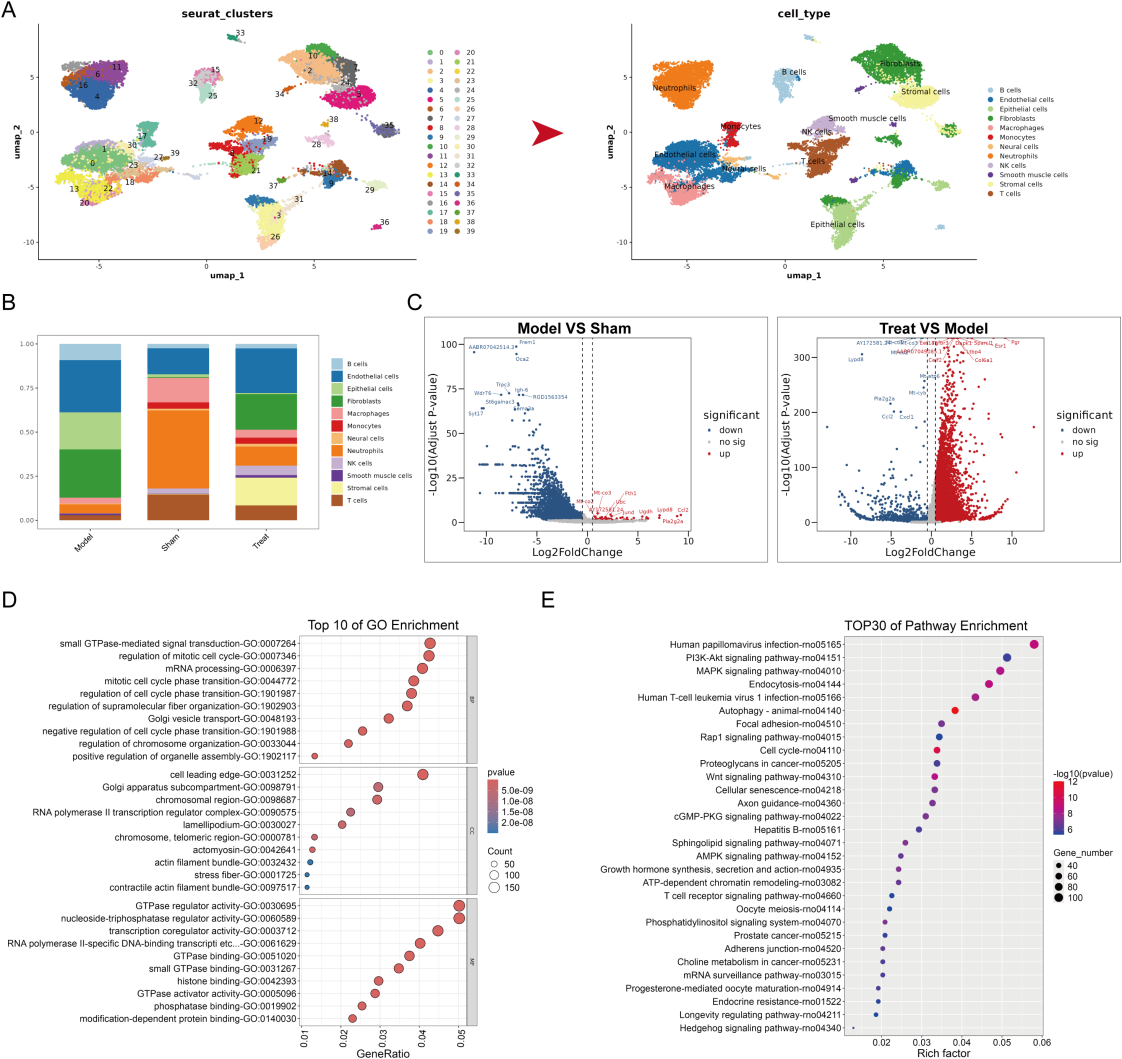
Single cell sequencing analysis. **(A)** Cell subgroup annotation, **(B)** Proportions of different subgroups in the three groups, **(C)** Volcano plot of differences in Fibroblasts, **(D)** GO enrichment analysis of Fibroblasts between Model and Sham group, **(E)** KEGG enrichment analysis of Fibroblasts between Model and Sham group.

To investigate the molecular mechanism by which Danshen injection ameliorates endometrial fibrosis in IUA rats, we performed scRNA-seq on uterine tissues from the Sham group, Model group, and Treat (Model+H-Danshen injection) group. A total of 22,378 high-quality cells were obtained. Through dimensionality reduction clustering and cell type annotation, 12 cell lineages were identified, including B cells, Endothelial cells, Epithelial cells, Fibroblasts, Macrophages, Monocytes, Neural cells, Neutrophils, NK cells, Smooth muscle cells, Stromal cells, and T cells (**Figure 3A**). Comparative analysis revealed that, compared with the Sham group, the proportion of fibroblasts was significantly increased in the Model group, while it was markedly downregulated after Danshen injection treatment (**Figure 3B**). These findings suggest that fibroblast expansion may be associated with IUA-related fibrotic remodeling and may represent a cellular component responsive to Danshen intervention.

In the differential volcano plot, compared with the Sham group, the Model group exhibited 3,765 downregulated genes and 51 upregulated genes. Compared with the Model group, the Treat group showed 1798 downregulated genes and 9118 upregulated genes. Notably, Sema3a, a key regulator of EMT, exhibited significant suppression in fibrotic tissues, suggesting its potential as a suppressive biomarker for endometrial repair mechanisms. Crucially, significant upregulation of Lamc2 and Cd44 was observed in the model group, whereas Danshen injection intervention demonstrated significant and robust downregulation of these markers. This reciprocal expression trajectory suggests that Danshen injection may exert its antifibrotic effects through the LAMC2-CD44 signaling axis. KEGG and GO enrichment analyses of differentially expressed genes in fibroblasts showed that they were enriched in signaling pathways related to fibrosis and cell signal transduction, such as Focal adhesion (**Figures 3D, E**), providing directions for further mechanistic research.

### 3.3 Cell to cell communication analysis

**Figure 4:**
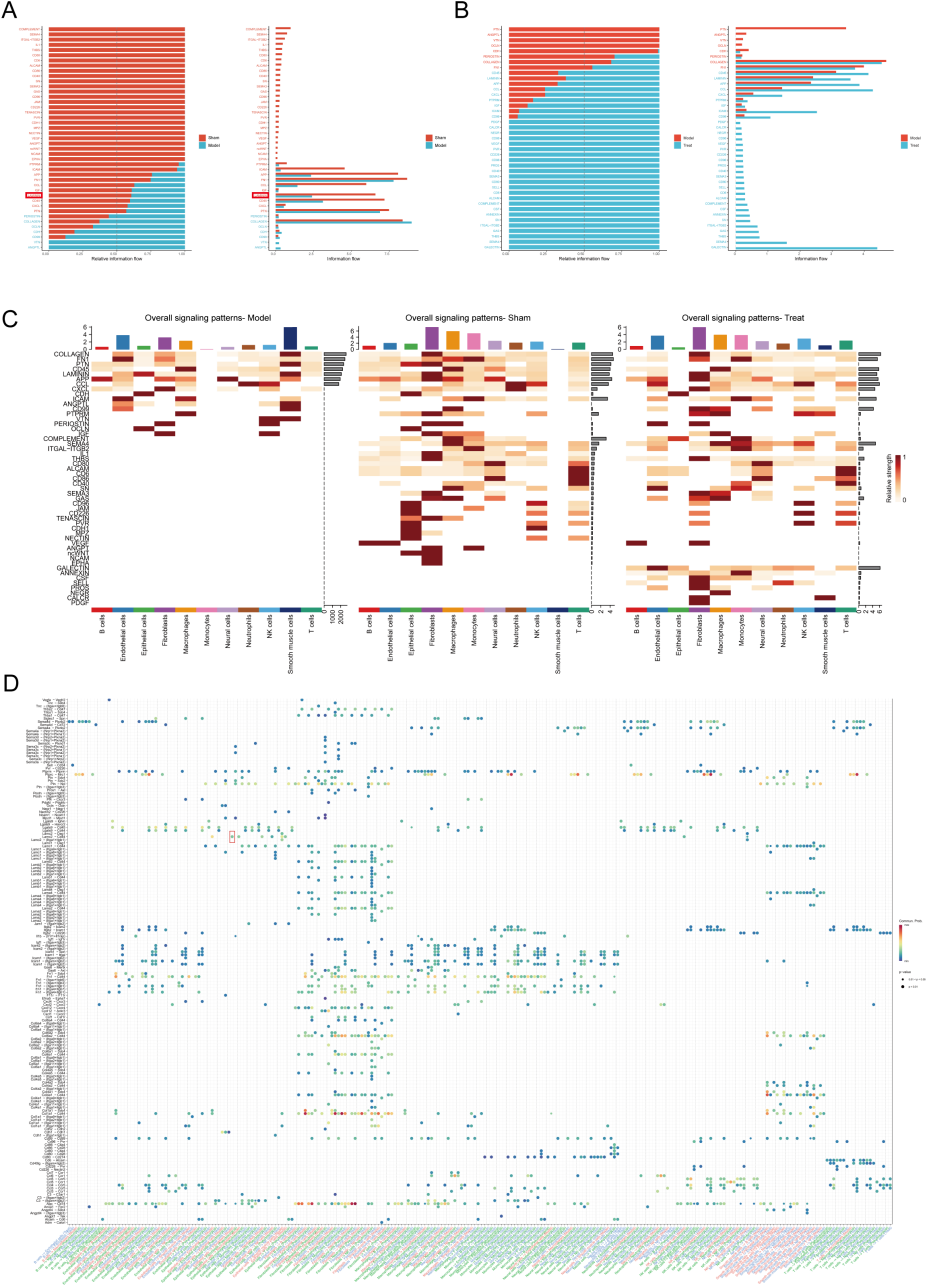
Cell to cell communication analysis. **(A)** Total information flow diagram of Model vs Sham, **(B)** Total information flow diagram of Treat vs Model, **(C)** Signaling pathway all heatmap, **(D)** All ligand receptor netVisual bubbleplot.

Global signaling information flow analysis (**Figure 4A**) revealed the disruption of the endometrial microenvironment during IUA progression. Comparative analysis between the Sham and Model groups demonstrated that under fibrotic conditions, signaling pathways critical for physiological tissue homeostasis and epithelial repair-including SEMA3, LAMININ, and ITGAL-ITGB2 networks-were significantly suppressed. Crucially, integration of the treated group revealed that administration of Danshen injection effectively reversed these pathological alterations. The treatment enhanced the activity of signaling pathways associated with tissue repair and homeostasis maintenance (**Figure 4B**).

To identify the specific cellular sources and molecular drivers of these fibrotic signals, we evaluated the global signaling patterns across all identified cell populations (**Figure 4C**). The Model group exhibited marked suppression of the LAMININ signaling network, whereas this suppression was significantly restored in both the Sham and treated groups, suggesting that Danshen may regulate IUA through the LAMININ signaling pathway. Further analysis of specific ligand-receptor pairs (**Figure 4D**) showed a significant increase in the interaction probability between fibroblast-derived LAMC2 and CD44 receptors on adjacent target cells, indicating that LAMC2-CD44 is possibly central to ECM deposition in IUA. Given that CD44 activation serves as an upstream activator of the TGF-β1/Smad cascade, these CellChat findings generated the hypothesis that Danshen injection ameliorates IUA not only by reducing fibroblast numbers but also by modulating the LAMC2-CD44 ligand-receptor network, thereby preventing downstream activation of profibrotic TGF-β1/SMAD2/3 signaling.

### 3.4 LPS induces activation of rat uterine fibroblasts

**Figure 5:**
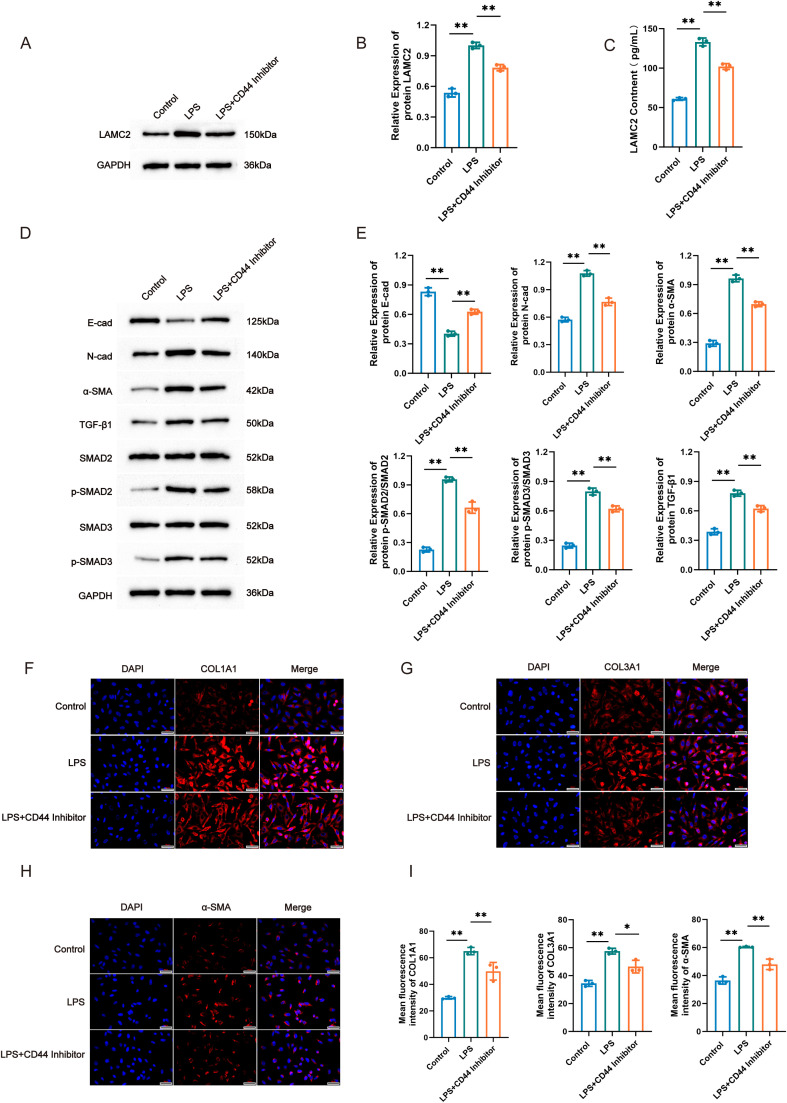
LPS induced activation of rat uterine fibroblasts. **(A)** WB detection of LAMC2 expression levels in epithelial cells, **(B)** Relative expression of LAMC2 in epithelial cells, **(C)** Level of LAMC2 in cells detected by ELISA in epithelial cells, **(D)** WB detection of E-cad, N-cad, α-SMA,TGF-β1, SMAD2/3 in fibroblasts, **(E)** Relative expression of E-cad, N-cad, α-SMA,TGF-β1, SMAD2, SMAD3 in fibroblasts, **(F)** Immunofluorescence staining of COL1A1 in fibroblasts, **(G)** Immunofluorescence staining of COL3A1 in fibroblasts, **(H)** Immunofluorescence staining of α-SMA in fibroblasts, **(I)** Mean fluorescence intensity of COL1A1, COL3A1 and α-SMA. Compared to model in fibroblasts, *P < 0.05, **P < 0.01.

To explore whether intercellular communication between endometrial fibroblasts and epithelial cells in rats via LAMC2-CD44 influences the progression of endometrial fibrosis, we examined the expression changes of ECM components, EMT markers, and key proteins in the TGF-β1/Smad signaling pathway. First, we assessed LAMC2 expression. WB results revealed that, compared with the Control group, the protein expression level of LAMC2 in endometrial epithelial cells of rats in the LPS-induced group was significantly elevated, while the addition of a CD44 inhibitor reversed LAMC2 expression (**Figures 5A, B**). Subsequently, ELISA was employed to detect the concentration of LAMC2 in the cell supernatant. It was found that LPS induction markedly promoted LAMC2 secretion, and the CD44 inhibitor effectively suppressed this effect (**Figure 5C**). These findings suggest that LPS can upregulate the expression and secretion of LAMC2 in a CD44-dependent manner. Next, we evaluated the impact of LPS on core markers of fibroblast activation and EMT-related proteins. WB analysis showed that after LPS induction, the expression of the epithelial marker E-cad was significantly downregulated, while the expressions of the mesenchymal markers N-cad and α-SMA were significantly upregulated. The addition of the CD44 inhibitor could significantly reverse the protein expression changes induced by LPS. To further explore the underlying molecular mechanism, we further examined the activity of the classic fibrosis-related TGF-β1/Smad pathway. The experimental data demonstrated that LPS exposure led to a marked upregulation of TGF-β1 protein expression and induced phosphorylation events in SMAD2/3, whereas total SMAD2/3 protein levels remained unchanged across the groups. Treatment with the CD44 inhibitor significantly inhibited the LPS-induced upregulation of TGF-β1 expression and the phosphorylation of SMAD2/3. These findings suggest that LPS drives fibroblast activation through TGF-β1/Smad pathway engagement, with this regulatory mechanism being critically dependent on CD44-mediated signaling (**Figures 5D, E**). Immunofluorescence staining results also confirmed that the fluorescence intensities of COL1A1, COL3A1, and α-SMA in fibroblasts were significantly enhanced after LPS induction (**Figures 5F–I**), further supporting the conclusion from a morphological perspective that LPS promotes fibroblast activation and extracellular matrix synthesis. In summary, *in vitro* experimental evidence demonstrates that LPS stimulation significantly induces alterations in LAMC2 expression levels, dynamic changes in EMT-related markers, and robust activation of the TGF-β1/SMAD signaling pathway in isolated rat uterine fibroblast populations. Notably, these intracellular specific responses can be partially reversed through pharmacological blockade of the CD44 receptor. This finding provides experimental support for the mechanistic hypothesis that LAMC2-CD44 interaction serves as a key upstream regulator of the profibrotic TGF-β1/SMAD signaling cascade during fibroblast intrinsic activation processes.

### 3.5 Danshen injection improves endometrial fibrosis in rats with intrauterine adhesions by inhibiting the LAMC2-CD44-TGF-β1- SMAD2/3 signaling pathway

**Figure 6:**
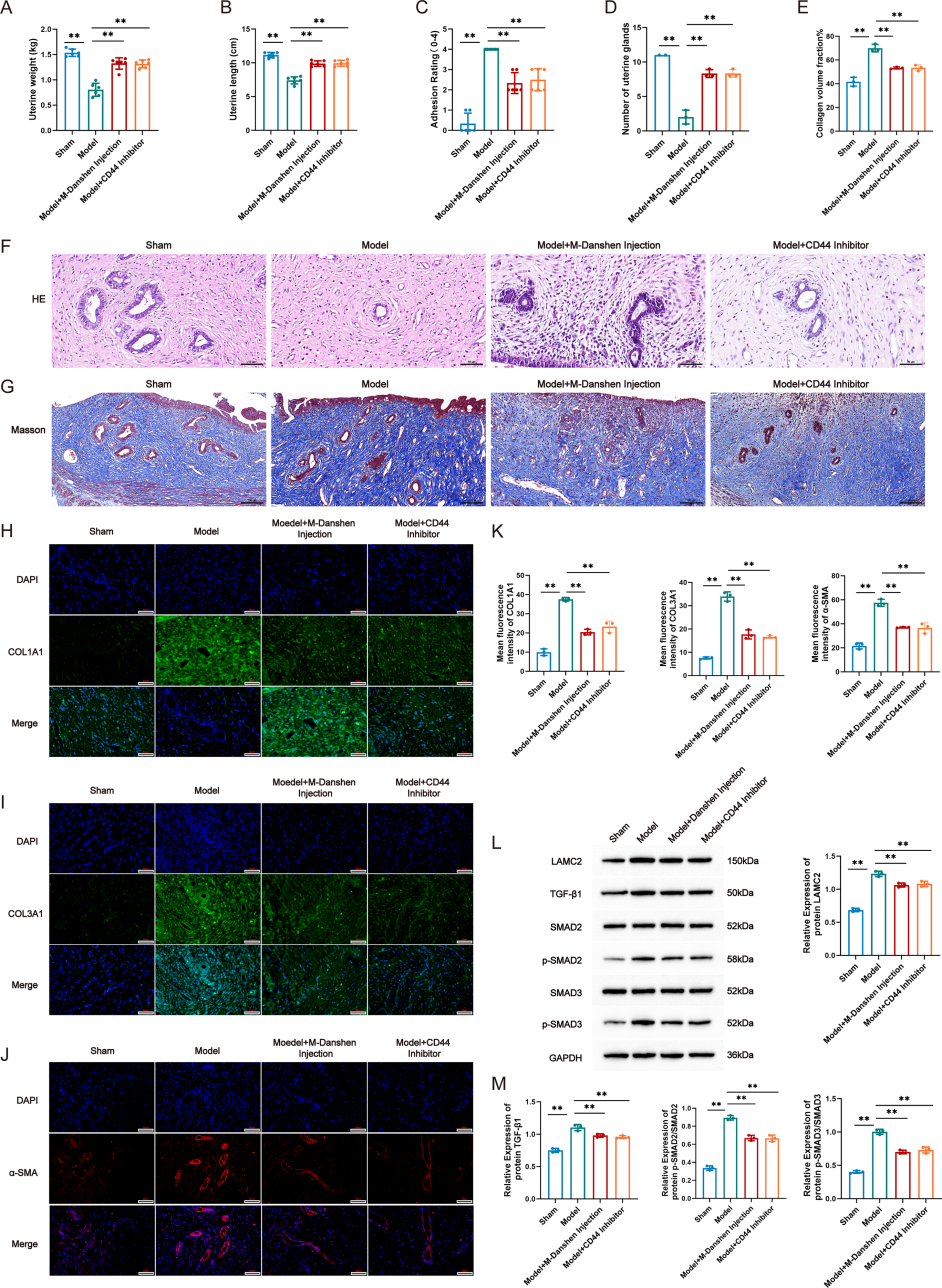
Danshen injection improves endometrial fibrosis in IUA rats by inhibiting the LAMC2-CD44-TGF-β1- SMAD2/3 signaling pathway. **(A)** Uterine Weight, **(B)** Uterine Length, **(C)** Grading of intrauterine adhesions in each group, **(D)**Number of uterine glands, **(E)** Collagen volume fraction, **(F)** H&E Staining, **(G)** MASSON Staining, **(H)** Immunofluorescence staining of COL1A1, **(I)** Immunofluorescence staining of COL3A1, **(J)** Immunofluorescence staining of α-SMA, **(K)** Mean fluorescence intensity of COL1A1, COL3A1 and α-SMA, L: WB detection of LAMC2, TGF-β1, SMAD2, SMAD3, **(L)** Relative expression of protein LAMC2, TGF-β1, SMAD2, SMAD3. Compared to model, *P < 0.05, **P < 0.01. For panels A-C, n = 6 independent animals per group; for panels **(D–M)**, n=3 independent animals per group.

To investigate whether Danshen injection exerts antifibrotic effects by regulating the LAMC2-CD44-TGF-β1- SMAD2/3 signaling pathway, we established an IUA model in rats and administered Danshen injection, and a CD44 inhibitor as interventions, respectively. The concentration screening of the CD44 inhibitor is shown in **Supplementary Figure 1**. First, we evaluated uterine morphology and histopathological changes. Compared with the Sham group, rats in the Model group exhibited a significant reduction in uterine length and weight, along with a marked increase in intrauterine adhesion grading. Both Danshen injection and CD44 inhibitor treatments effectively reversed these trends (**Figures 6A–C**). Histological staining analysis further confirmed these findings: H&E staining revealed a significant decrease in the number of endometrial glands in the Model group, while gland numbers increased significantly after Danshen injection and CD44 inhibitor treatments (**Figures 6D, F**). Masson staining indicated extensive abnormal collagen fiber deposition and a significantly elevated percentage of fibrotic area in the endometrium of the Model group. In contrast, collagen deposition and fibrotic area were markedly reduced following Danshen injection and CD44 inhibitor interventions (**Figures 6E, G**). To clarify the expression changes of fibrosis-related proteins, we detected the expressions of COL1A1, COL3A1, and α-smooth muscle actin (α-SMA) via immunofluorescence. The results showed that, compared with the Sham group, the fluorescence intensities of COL1A1, COL3A1, and α-SMA were significantly enhanced in the Model group. However, their expressions were effectively suppressed after Danshen injection and CD44 inhibitor treatments (**Figures 6H–K**). Finally, to explore the underlying molecular mechanism, we examined the expressions of key molecules in the LAMC2-CD44-TGF-β1-SMAD2/3 signaling pathway. WB results demonstrated that, compared with the Sham group, the protein levels of LAMC2, TGF-β1, p-SMAD2/SMAD2, and p-SMAD3/SMAD3 in uterine tissues were significantly upregulated in the Model group. After Danshen injection and CD44 inhibitor treatments, the expressions of these key signaling molecules were effectively suppressed (**Figures 6L, M**). The above results indicate that Danshen injection can effectively ameliorate endometrial fibrosis in IUA rats, with effects comparable to those of the CD44 inhibitor. Mechanistically, Danshen injection may exert antifibrotic effects by inhibiting the interaction between LAMC2 and CD44, thereby impeding the activation of the downstream TGF-β1- SMAD2/3 signaling pathway.

## 4 Discussion

Danshen injection, a TCM preparation with blood-activating and stasis-resolving as well as anticoagulant properties, is widely used in the clinical treatment of diseases related to impaired blood circulation (Zhang et al., 2022). Its major active components exhibit anti-inflammatory, antifibrotic, and tissue-repair potentials, indicating multi-target pharmacological effects in mitigating fibrotic diseases (Yang L. et al., 2025). involves pathological mechanisms including inflammation, epithelial-mesenchymal transition (EMT), and extracellular matrix (ECM) accumulation forming a complex fibrotic network (Qin et al., 2025). Fibrosis alters endometrial structure and function, creating abnormal implantation environments that lead to embryo implantation failure. Compared to other drugs or materials for IUA prevention/treatment, Danshen injection demonstrates advantages of broad availability, low cost, and fewer adverse reactions. In postoperative IUA management, *in vitro* studies of *Salvia miltiorrhiza* extracts have shown inhibition of TGF-β1-induced fibroblast activation, thereby reducing fibrogenesis (Hsu et al., 2024). Verma et al. (2023) identified that tanshinone IIA and other Danshen injection components exhibit signal modulation and antifibrotic effects across multiple fibrosis models, further supporting the mechanistic rationale of SMI in this study.

TGF-β serves as a pivotal mediator of fibrosis, primarily inducing fibrotic processes through the Smad-dependent pathway, and has been confirmed as a core regulator of fibrosis in multiple studies (Dong et al., 2025; Yuan et al., 2025; Di et al., 2025). Pathological fibrosis in IUA is closely associated with EMT, where TGF-β1/Smad2/3 signaling activation promotes epithelial-to-mesenchymal transition and stimulates ECM accumulation, driving endometrial fibrotic progression (Zhao et al., 2024; Wang et al., 2023). Lamc2 is an ECM protein involved in cell adhesion and migration, regulating tissue structural stability in fibrotic conditions (Tong et al., 2024). CD44 is a transmembrane cell surface glycoprotein that binds to ECM components and participates in signal transduction; its inhibition can reduce the expression of fibrosis-related proteins (Guo et al., 2024). Although previous studies have indicated that TGF-β, Lamc2, and CD44 are all involved in fibrosis, their interactions in endometrial fibrosis of IUA remain to be elucidated. This study focuses on the Lamc2-CD44-TGF-β1/Smad2/3 axis and investigates its role in the antifibrotic effects of Danshen injection.

In this study, a rat IUA model was established to systematically investigate the molecular mechanism by which Danshen injection ameliorates endometrial fibrosis through regulation of the Lamc2-CD44-TGF-β1/Smad2/3 signaling pathway. The results demonstrated that Danshen injection significantly improved uterine structural damage in IUA rats, reduced collagen deposition, and modulated changes in EMT-related markers (e.g., upregulation of CK-19 and downregulation of Vimentin). The core mechanism involves attenuation of Lamc2-CD44-associated signaling, thereby inhibiting activation of the downstream TGF-β1/Smad2/3 pathway, which in turn reduces fibroblast activation and ECM deposition (e.g., decreased expression of Col1α1, Col3α, and α-SMA). This finding is consistent with the study showing that Tanshinone IIA ameliorates fibrosis in endometriosis by inhibiting the TGF-β/Smad pathway (Ma et al., 2021) and shares similarities with the mechanism by which Bushen Huoxue Decoction inhibits EMT via regulation of the miR-665/CIRBP pathway (Feng et al., 2024). Single-cell sequencing revealed a significant increase in the proportion of fibroblasts in the IUA model group, with enrichment in fibrosis-related pathways such as focal adhesion. Further intercellular communication analysis confirmed that Lamc2, as an ECM component, participates in altered signaling patterns in IUA via the Lamc2-CD44 axis and activates the TGF-β1/Smad2/3 pathway. *In vitro* experiments further validated that LPS-induced fibroblast activation was accompanied by increased Lamc2 expression and CD44-dependent secretion of TGF-β1; CD44 inhibitors significantly suppressed Smad2/3 phosphorylation and reduced expression of fibrotic markers, supporting the role of CD44 as an upstream regulator of Lamc2-associated signaling. Animal experiments confirmed that Danshen injection downregulated Lamc2 and CD44 expression while inhibiting Smad2/3 phosphorylation, providing evidence that its antifibrotic effects are mediated through interference with the Lamc2-CD44 axis.

Although this study has preliminarily elucidated the molecular mechanism by which Danshen injection ameliorates endometrial fibrosis in IUA through the LAMC2-CD44-TGF-β1-SMAD2/3 signaling pathway, several limitations remain to be further explored. Firstly, as a multi-component compound preparation, the specific active ingredients in Danshen injection on the LAMC2-CD44 axis are still unclear. It is necessary to clarify the key pharmacodynamic material basis through component separation and functional verification. Secondly, the changes in the signaling pathway observed in animal models differ from the pathological microenvironment of human IUA. Therefore, it is essential to validate the expression profile and spatial distribution characteristics of LAMC2-CD44-TGF-β1-SMAD2/3 in the endometrium of patients using clinical samples. Finally, there is a lack of follow-up data on the long-term effects of Danshen injection on the reversal of EMT and the restoration of fertility, and the potential mechanism by which it regulates stem cell properties has not yet been elucidated.

This study provides evidence that Danshen injection ameliorates endometrial fibrosis in IUA in association with reduced LAMC2-CD44-related signaling and attenuation of TGF-β1-SMAD2/3 pathway activation, and supports a potential multi-target synergistic effect of Danshen injection. This research enhances our comprehensive understanding of the molecular regulatory processes of EMT in uterine epithelial cells. As our comprehension of EMT deepens and its molecular regulatory mechanisms become more thoroughly understood, interfering with the EMT process through various techniques and strategies may offer novel therapeutic approaches for addressing intrauterine adhesions.

## Data Availability

The data are available from the corresponding author on reasonable request.
