# Supplementary figures and images for: Molecular mechanism of Danshen injection in treating endometrial fibrosis induced by intrauterine adhesions via the LAMC2-CD44-TGF-β1-SMAD2/3 signaling pathway
Source: Front Physiol. 2026 Apr 10;17:1794215. doi: 10.3389/fphys.2026.1794215 (PMC13106170; doi:10.3389/fphys.2026.1794215)

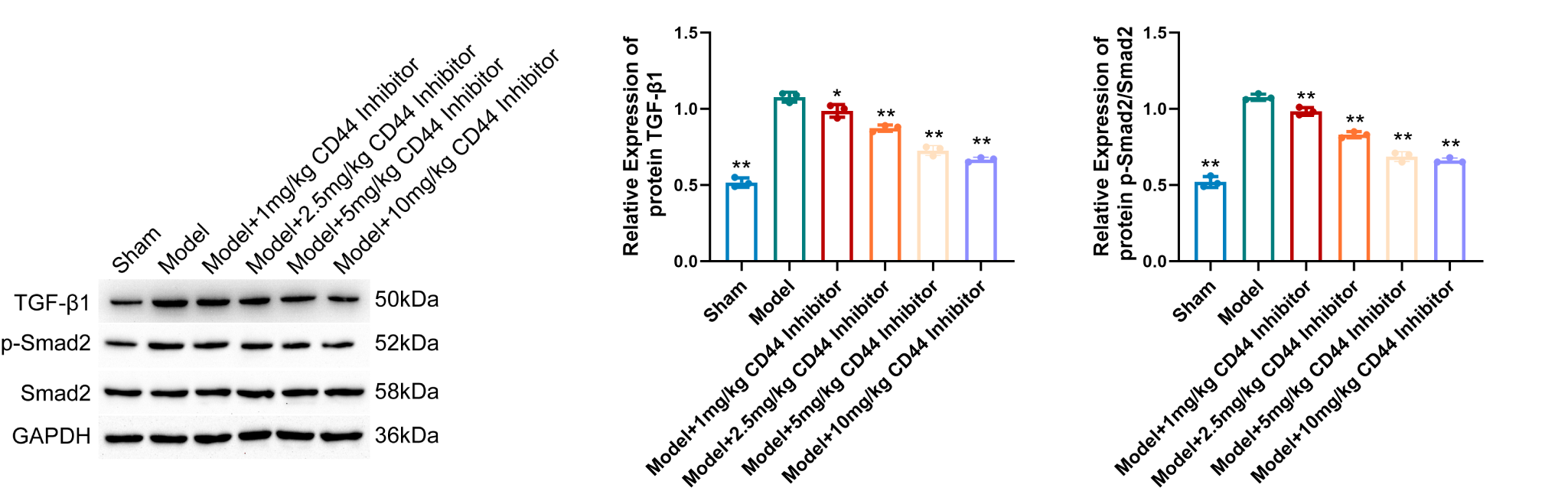

Supplement: Supplementary file 1 [file Image1.tif]
